# Supplementary material for: Single cell RNA-sequencing identifies a metabolic aspect of apoptosis in Rbf mutant
Source: Nat Commun. 2018 Nov 27;9:5024. doi: 10.1038/s41467-018-07540-z (PMC6258665; doi:10.1038/s41467-018-07540-z)
Supplement: Supplementary file 1 — Supplementary Information [file 41467_2018_7540_MOESM1_ESM.pdf]

Single cell RNA-sequencing identifies a metabolic aspect of apoptosis in *Rbf* mutant

Majd M. Ariss et. al

Supplementary Figure 1. Cell of origin and R type feature plots

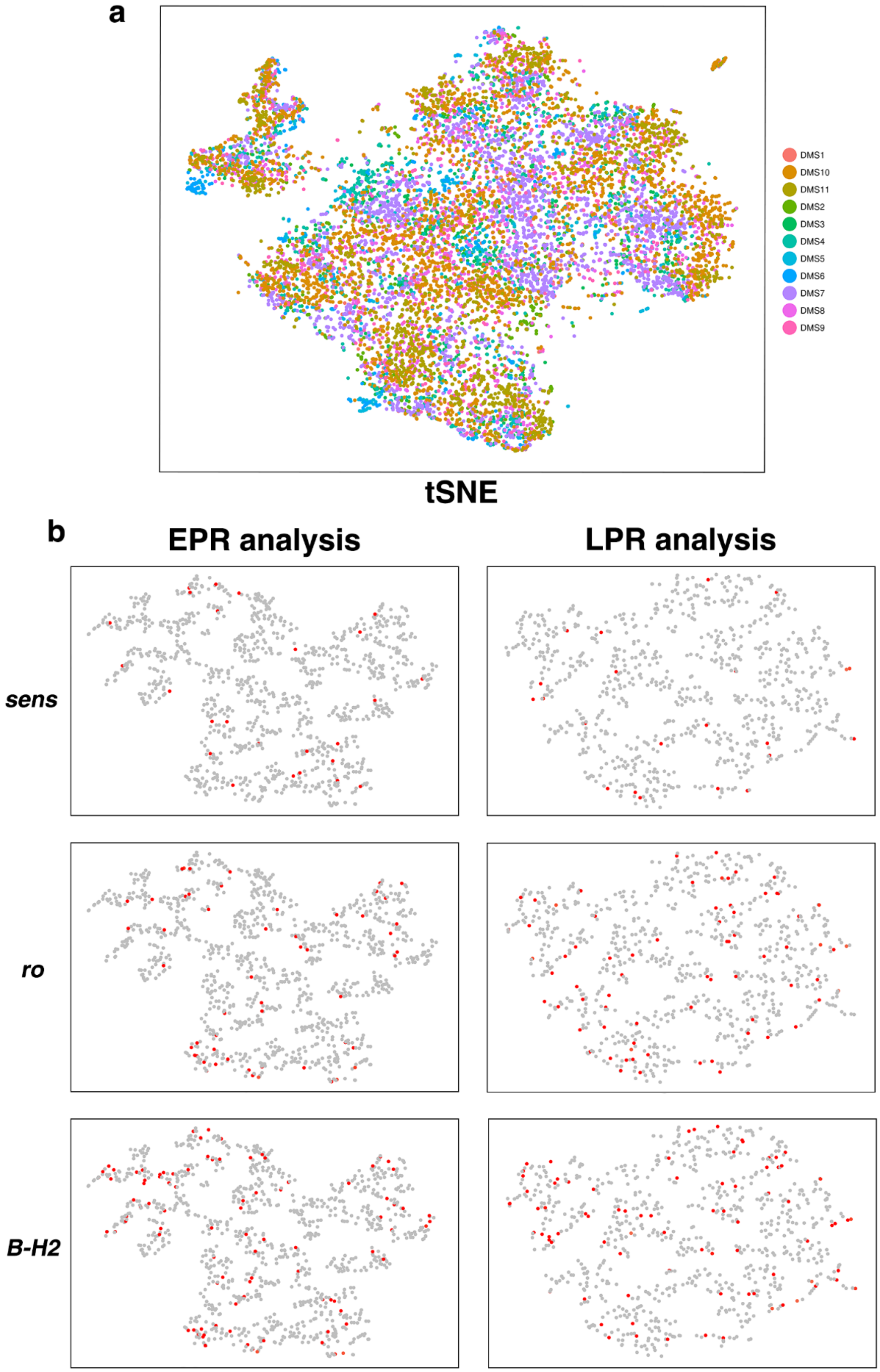

a) tSNE showing the populations shared by all replicates (apart from HEMO which is shared by 10 replicates). Legend: DMS # = Drosophila Melanogaster Sample #. b) Left panel: Feature plots for *sens*, *ro*, and *B-H2* following Seurat analysis on EPR cells. Right panel: Feature plots for *sens*, *ro*, and *B-H2* following Seurat analysis on LPR cells. Both panels show randomly scattered expression of *sens*, *ro*, and *B-H2* indicating that photoreceptors do not cluster by R-type within EPR and LPR.

Supplementary Figure 2. Monocle 2 trajectory using UND PPN MF INT EPR LPR

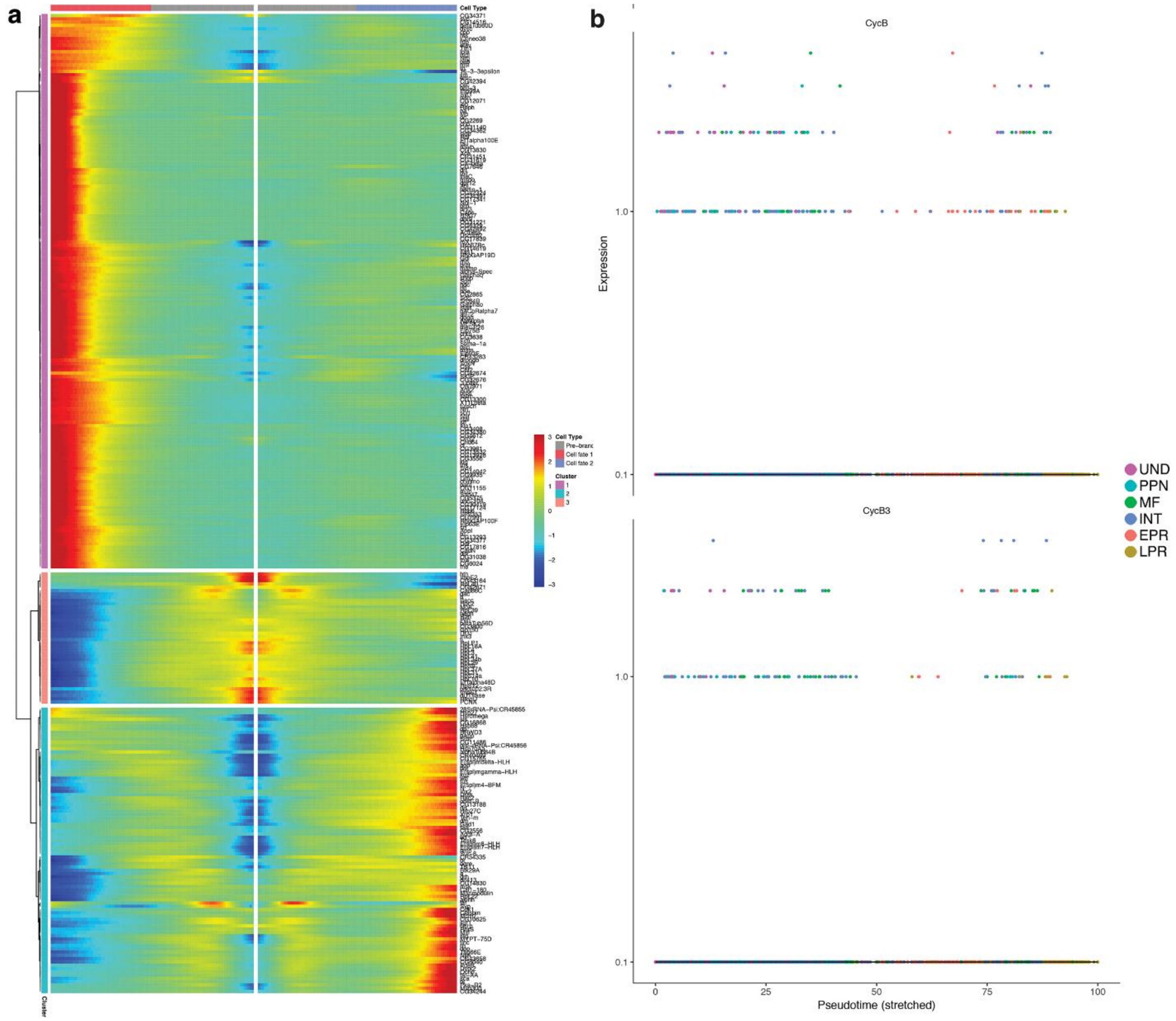

a) Heatmap showing marker highly expressed in each branch. b) CycB and B3 are expressed in INT cells indicating that the middle top branch in the pseudotime is due to G2/M arrested INT cells.

Supplementary Figure 3. Activation of apoptosis does not result in low pH

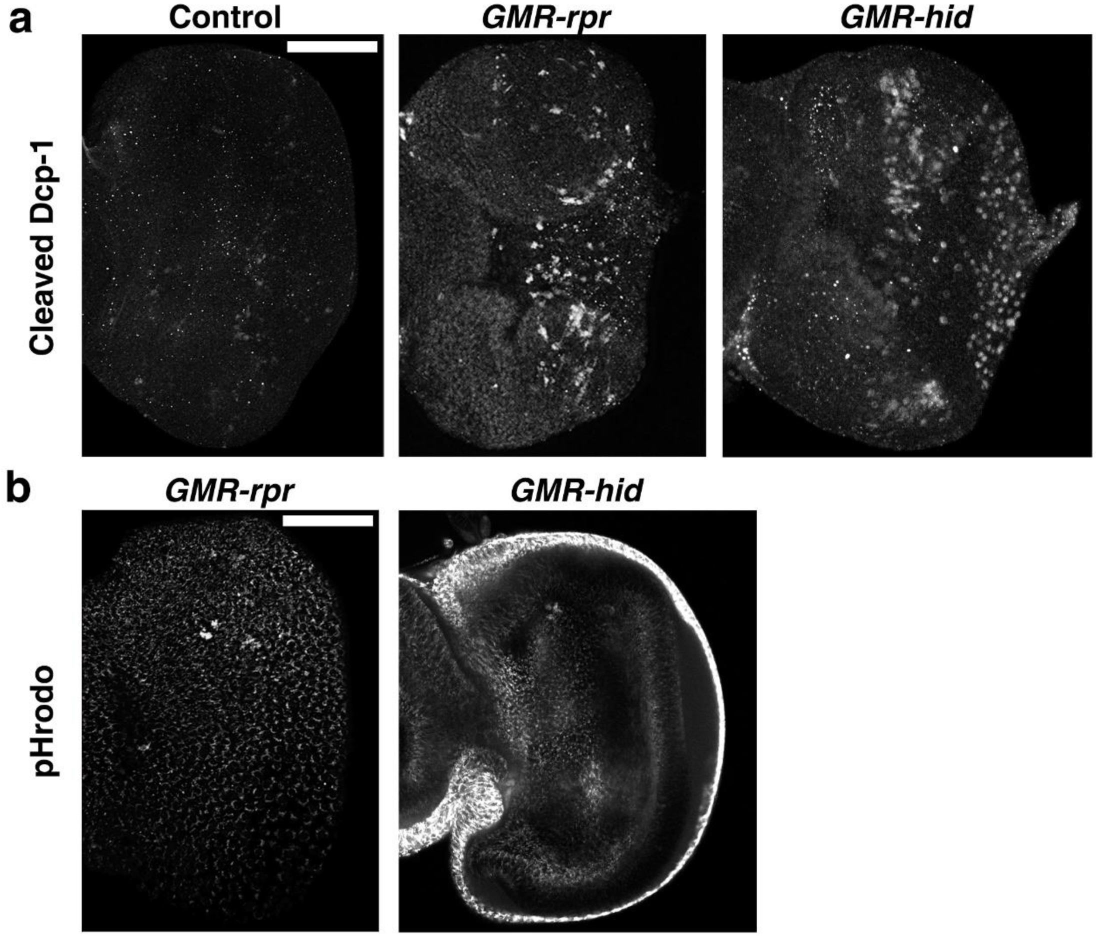

a) Cleaved Dcp-1 staining shows an increase in apoptosis in *GMR-rpr* and *GMR-hid* eye discs, compared to control. The scale bar is 100 µm. b) No reduction in pH was observed in *GMR-rpr* and *GMR-hid* eye discs. The scale bar is 100 µm.

Supplementary Table 1. Cell type marker list for labelling tSNE

**Population**	**Genes**	**Note**
ANT	*Dll, disco-r, ss, hth*	Does not express Lim1
EAB	*ct, Lim1, hth*	Does not express Dll
DPE	*mirr, hth*	Shares genes with Ocx
OCx	*oc, hth*	Shares genes with DPE
VPE	*cv-c, hth*	Does not express *mirr, ct, Lim1* or *oc*
UND	*hth, toy*	Does not express *Lim1*, *ct*, *h*, and *dac*
PPN	*dac, h*	PPN loses expression of *hth* vs. UND
MF	*E(spl)* genes, *Notch, dac*	Does not express *h*
INT	*ed, Mmp2*	Does not express neuronal markers or cell cycle genes
SMW	*Claspin, PCNA, Mcm7, Mcm10, stg, dap*	Cell cycle genes are upregulated. Shares markers with INT and does not express neuronal markers
EPR	*twit, scrt, Futsch, Appl, elav*	Expressed neuronal markers and shares markers with INT
LPR	*jeb, nrv3, nSyb, Ace, Dscam2*	Expresses neuronal markers and shares markers with INT. Top genes play a role in axonogenesis
PG	*Con, stg, CG3168, CG9328*	Repo and cell cycle genes are upregulated
WG+SPG	*gli, nrv2, sty, moody*	Repo is upregulated
HEMO	*hml, BM-40-SPARC, Cg25C, regucalcin*	

Supplementary Table 2. Box plot values of Dcp-1 quantified eye discs

	WT	*Rbf*^{120a}	*Rbf*^{120a}, *Ldh*^{RNAi}	*Rbf*^{120a}, *Ald*^{RNAi}	*Rbf*^{120a}, *HIF1A*^{RNAi}
Minimum	9	63	28	23	29
First Quartile	22.0	173.2	118.2	78.25	78.0
Median	39.0	219.5	158.5	129.0	116.0
Third Quartile	61.0	254.8	181.5	170.25	157.0
Maximum	338	382	325	313	282

Supplementary Table 3. ChIP-PCR values using Rbf, E2F1, Dp and E2F2 antibodies

**Enrichment relative to negative site**					
	IgG	Rbf	E2F1	Dp	E2F2
**Negative site**	1	1	1	1	1
Ald	1.301549195	63.68792833	4.410761131	47.1136544	85.13083984
HIF1A	1.410430408	103.0747969	4.628343388	69.85083046	134.0208244
**Standard error**					
	IgG	Rbf	E2F1	Dp	E2F2
**Negative site**	0.05237828	0.088868346	0.121753486	0.033081019	0.107810003
Ald	0.051681838	1.732083308	0.489779534	0.204007254	3.879614175
HIF1A	0.258034781	0.11153005	0.299901351	1.261072236	2.578694796
