# Supplementary material for: Single cell RNA-sequencing identifies a metabolic aspect of apoptosis in Rbf mutant
Source: Nat Commun. 2018 Nov 27;9:5024. doi: 10.1038/s41467-018-07540-z (PMC6258665; doi:10.1038/s41467-018-07540-z)
Supplement: Supplementary file 2 — Description of Additional Supplementary Files [file 41467_2018_7540_MOESM2_ESM.docx]

**Title:** Supplementary Data 1.
**Description:** Differentially expressed genes in each population in the WT analysis

**Title:** Supplementary Data 2.
**Description:** Average expression of genes in the WT analysis

**Title:** Supplementary Data 3.
**Description:** GOBP for UND vs. EPR and LPR

**Title:** Supplementary Data 4.
 **Description:** GOBP for EPR vs. LPR and UND

**Title:** Supplementary Data 5.
**Description:** GOBP for LPR vs. EPR and UND

**Title:** Supplementary Data 6.
**Description:** Differentially expressed genes in each population in the WT and Rbf120a analysis g.

**Title:** Supplementary Data 7.
**Description:** Average expression of genes in the WT and Rbf120a analysis h.

**Title:** Supplementary Data 8.
**Description:** Cell barcodes used in the WT analysis i. Supplementary Data 9. Cell barcodes used in the WT and Rbf120a analysis

**Title**: Supplementary Data 9.

**Description**: Cell barcodes used in the WT and Rbf120a analysis
