# Supplementary material for: Single cell RNA-sequencing identifies a metabolic aspect of apoptosis in Rbf mutant
Source: Nat Commun. 2018 Nov 27;9:5024. doi: 10.1038/s41467-018-07540-z (PMC6258665; doi:10.1038/s41467-018-07540-z)
Supplement: Supplementary file 14 — Source Data [file 41467_2018_7540_MOESM14_ESM.docx]

**Title:** Source Data

**Description:** Unprocessed Data for Figure 1a, Figure 1b, Supplementary Table 2 and Supplementary Table 3
